# Strong interface-induced spin–orbit interaction in graphene on WS_2_

**DOI:** 10.1038/ncomms9339

**Published:** 2015-09-22

**Authors:** Zhe Wang, Dong–Keun Ki, Hua Chen, Helmuth Berger, Allan H. MacDonald, Alberto F. Morpurgo

**Affiliations:** 1 Department of Quantum Matter Physics (DQMP) and Group of Applied Physics (GAP), University of Geneva, 24 Quai Ernest–Ansermet, CH1211 Genéve 4, Switzerland; 2 Department of Physics, The University of Texas at Austin, Austin, Texas 78712, USA; 3 Institut de Physique de la Matière Complexe, Ecole Polytechnique Federale de Lausanne, CH–1015 Lausanne, Switzerland

## Abstract

Interfacial interactions allow the electronic properties of graphene to be modified, as recently demonstrated by the appearance of satellite Dirac cones in graphene on hexagonal boron nitride substrates. Ongoing research strives to explore interfacial interactions with other materials to engineer targeted electronic properties. Here we show that with a tungsten disulfide (WS_2_) substrate, the strength of the spin–orbit interaction (SOI) in graphene is very strongly enhanced. The induced SOI leads to a pronounced low-temperature weak anti-localization effect and to a spin-relaxation time two to three orders of magnitude smaller than in graphene on conventional substrates. To interpret our findings we have performed first-principle electronic structure calculations, which confirm that carriers in graphene on WS_2_ experience a strong SOI and allow us to extract a spin-dependent low-energy effective Hamiltonian. Our analysis shows that the use of WS_2_ substrates opens a possible new route to access topological states of matter in graphene-based systems.

## Abstract (web summary)

Routes towards inducing strong spin–orbit coupling in graphene have been hindered by detrimental effects on its electronic properties and material quality. Here, the authors demonstrate a possible solution by exploiting interfacial interactions between graphene and a tungsten disulfide substrate.

## Graphical abstract

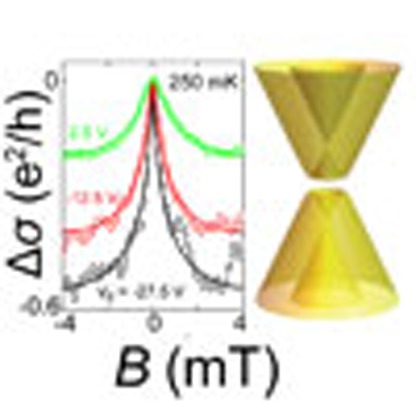

Because of the Dirac nature of its charge carriers and the presence of two valleys, graphene is a two-dimensional topological insulator^1^,^2^. Topological state characteristics have not been observed experimentally, because the strength of the spin–orbit interaction (SOI) intrinsically present in graphene is too weak^3^,^4^,^5^. Various strategies to amplify the SOI strength have been proposed theoretically^6–8^ or explored experimentally^11^. However, increasing the SOI strength in graphene without drastically affecting other basic aspects of its electronic structure, or the material quality, is proving extremely difficult^11^. Here we explore whether it is possible to induce strong SOI while preserving the quality of graphene, by exploiting interfacial interactions^12^,^13^,^14^ at an atomically sharp interface between graphene and a semiconducting WS_2_ crystalline substrate.

Many semiconducting transition metal dichalcogenides, such as WS_2_, are ideal substrates for graphene. Like hexagonal boron nitride (hBN)^15^, they are atomically flat and chemically inert, which is key to preserving high-quality transport properties (mobility values as high as *μ*∼50,000–60,000 cm^2^ V^–1^ s^–1^ have been recently reported for graphene on WS_2_) (ref. 16). Transition metal dichalcogenide crystals consist of a stack of monolayers having a hexagonal lattice that—like graphene—leads to the presence of two valleys in their electronic structure at the K and K′ point of the Brillouin zone^17^. The SOI in WS_2_ is extremely strong—several hundreds of millivolts in the valence bands and several tens of millivolts in the conduction band—and in monolayers it pins spin to valley^18^,^19^,^20^. The spins of states near the band edges point in one direction in one of the valleys and in the opposite direction in the other, a behaviour resembling the one expected theoretically in disorder-free graphene^1^,^2^. The ability of this substrate material to induce a strong SOI in graphene—as well as the nature of the induced SOI—is, therefore, an important topic that has attracted recent attention^10^.

Here we address these issues by combining a study of low-temperature quantum transport in graphene-on-WS_2_ devices with *ab initio* electronic structure calculations. Specifically, we perform systematic magnetotransport measurements to show that when transferred onto WS_2_ substrates graphene exhibits a pronounced and robust weak anti-localization (WAL) effect throughout the explored carrier density and temperature range, down to 250 mK. The detection of WAL provides a direct demonstration of the SOI enhancement in graphene due to the interfacial interactions with WS_2_ substrate. In the attempt to estimate quantitatively the magnitude of SOI enhancement, we show that the magnetotransport data can be fit to the theory of WAL for graphene in the presence of SOI, from which we determine the spin-relaxation time (*τ*_so_). We find that the value of *τ*_so_ (∼2.5–5 ps) in graphene on WS_2_ is 100–1,000 times shorter than *τ*_so_ in pristine graphene on SiO_2_ or hBN. This very strong enhancement of SOI found experimentally is consistent with the result of our *ab initio* calculations, which indicate that hybridization with the WS_2_ substrate orbitals is responsible for the SOI induced in graphene, and estimate the SOI strength under the conditions of the experiments to be ∼5 meV. Finally, we show that the results of our calculations close to the K and K′ points can be mapped onto a long-wavelength effective Hamiltonian, which, depending on the values of the parameter, describes a topologically insulating state. We therefore conclude that the possibility of using interfacial interactions to induce a strong SOI in graphene while preserving the high quality of the material opens a new possible route to create and investigate a topological insulating state in graphene.

## Results

### High quality of graphene-on-WS_2_ device

**Figure 1:**
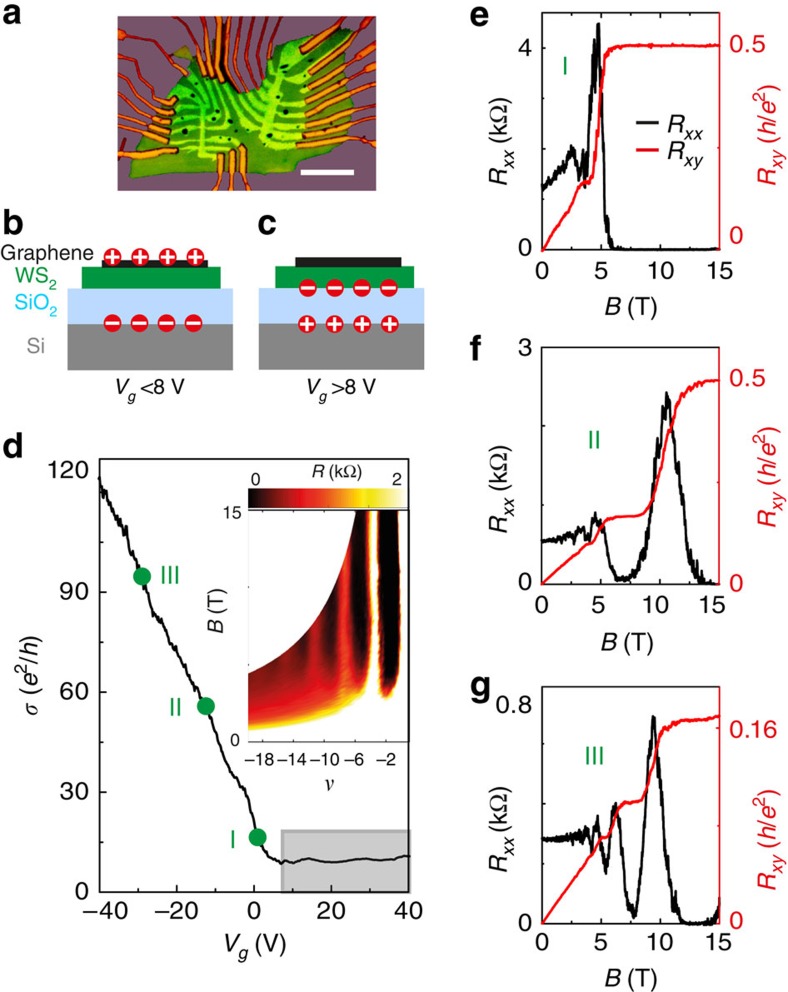
Basic electrical transport properties of graphene-on-WS_2_ heterostructure at 250 mK. (**a**) Optical microscope image of one of our devices, with multiple contacts in a Hall-bar geometry (scale bar, 10 μm). (**b**,**c**) Schematic cross-section of the device illustrating where charge is accumulated on varying *V*_*g*_ below and above ∼8 V (**b** and **c**, respectively). Owing to the presence of the n-doped WS_2_ substrate, charges are accumulated in graphene only for *V*_*g*_ lower than ∼8 V (the precise value slightly varies from sample to sample, depending on the doping level of the WS_2_ substrate.). (**d**) The conductivity (*σ*) of the device varies linearly with *V*_*g*_, below ∼8 V, and saturates for larger *V*_*g*_ values (shadow area). The green dots mark the ranges of *V*_*g*_ (I: 0–5 V; II: −10 to −15 V; and III: −25 to −30 V) used to perform ensemble averages of the device magnetoconductance. (The inset shows Shubnikov-de Hass oscillation in the longitudinal resistance *R*_*xx*_ originating from the half-integer quantum-Hall effect characteristic of Dirac fermions, with the black regions corresponding to *R*_*xx*_ minima that occur at values of filling factor |*ν*|=|*nh/eB*|*=*4 × (*N*+1/2), with *N* being integer) (**e**–**g**) Fully developed half-integer quantum-Hall effect with vanishing *R*_*xx*_ (black curve) and quantized *R*_*xy*_ (red curve) observed at different values of *V*_*g*_=0, −11.5 and −28 V (from **e**–**g**) within the region I, II and III (indicated in **d**).

We start by characterizing the basic transport properties of the graphene-on-WS_2_ devices used in our experiments. The devices are assembled in a multi–terminal Hall bar configuration, placed on a highly doped Si wafer that acts as a gate electrode and is coated with 280 nm SiO_2_ (see Fig. 1a for an optical microscope image, Fig. 1b,c for a schematic of the device structure and the Methods section for the details of the device fabrication). We have realized several such devices. Here we present representative data from one of them (similar data from another device can be found in the Supplementary Note 1 and Supplementary Fig. 1). Figure 1d shows that on ramping up the gate voltage from *V*_*g*_=–40 V, the conductivity *σ* of graphene on WS_2_ decreases linearly until *V*_*g*_∼8 V, after which it saturates. Saturation occurs because, for *V*_*g*_>8 V, electrons are accumulated at the interface between the SiO_2_ and the WS_2_ crystal, screening the effect of the gate on the graphene layer on top (Fig. 1c, Supplementary Fig. 2 and Supplementary Note 2)^21^. (Because the mobility of charge carriers in WS_2_ is much smaller than in graphene^22^, carriers at the WS_2_/SiO_2_ interface give a negligible contribution to transport.) Sweeping the gate voltage down for *V*_*g*_<8 V, on the contrary, results in an increase (Fig. 1b) of carrier (hole) density in graphene and the conductivity increases. In our devices, therefore, the position of the Fermi level can be gate shifted in the graphene valence band, but accumulation of electrons at the SiO_2_/WS_2_ interface prevents access to the conduction band. In the following we therefore study only hole transport through graphene. To illustrate this conclusion, and to start assessing the device quality, Fig. 1e–g shows that the half-integer quantum Hall effect characteristic of monolayer graphene^23^ is clearly observed for different values of *V*_*g*_ between –40 and 8 V. The longitudinal resistance measured in this *V*_*g*_ range, and plotted versus filling factor *ν* and magnetic field *B* (the inset of Fig. 1d), confirms this result. We estimate the carrier mobility from *σ* using the hole density *n* extracted from the (classical) Hall effect and by looking at the slope d*σ/*d*V*_*g*_, and obtain in both cases *μ*∼13,000 cm^2^ V^–1^ s^–1^ at *T=*4.2 K. Since no effort has yet been put into optimizing the fabrication process, these values confirm the very good quality of graphene-on-WS_2_ devices found in earlier work^16^.

### Robust low-temperature WAL reveals strong SOI

**Figure 2:**
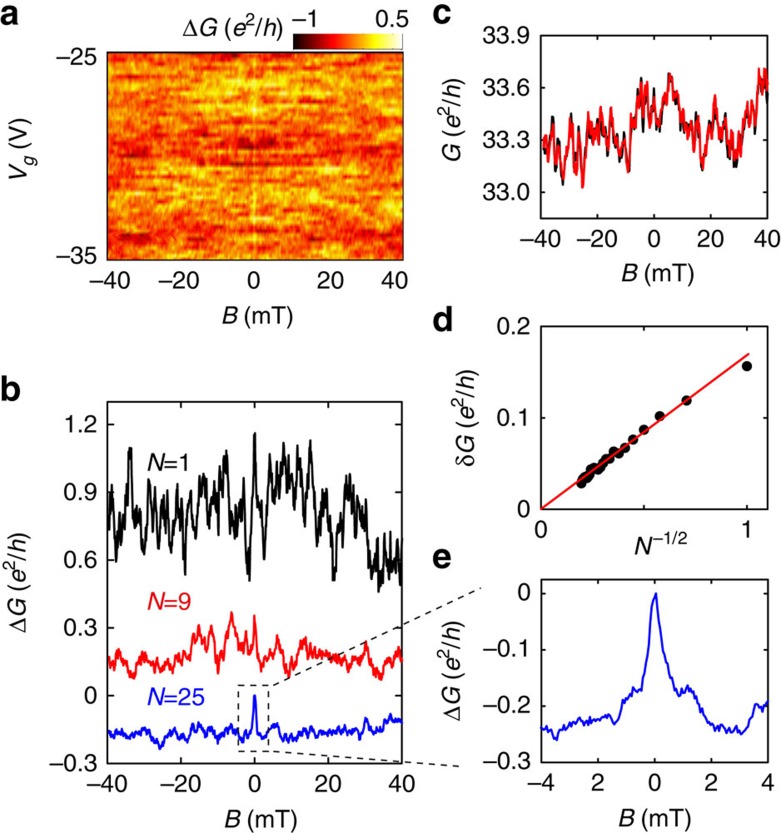
Ensemble averaging of the MC at 250 mK. (**a**) Colour-coded MC, Δ*G*(*B*), as a function of *V*_*g*_, with the background conductance slowly varying in *V*_*g*_ subtracted. The large background conductance fluctuations originating from phase-coherent interference of electron waves are apparent, and nearly completely obscure the effect of WAL around *B=0 *T. (**b**) Evolution of the averaged MC on increasing the number *N* of uncorrelated MC traces used to calculate the average (*N*=1, 9 and 25; curves offset for clarity): the conductance peak at zero *B* associated to WAL becomes apparent for sufficiently large values of *N*. (**c**) Two MC traces measured at *V*_*g*_=−25 V (red and black curves) demonstrating the excellent reproducibility of the conductance fluctuations. (**d**) After averaging over *N* different curves, the root mean square amplitude of the conductance fluctuations (δ*G*) decreases proportionally to *N*^−1/2^ as expected for a proper ensemble-averaging process. (**e**) Zoomed-in view of the ensemble-averaged MC (for *N=25*), which clearly exhibits a sharp conductance peak at *B=0 *T.

To demonstrate the presence of SOI in our devices, we probe WAL, which usually manifests itself as a characteristic sharp magnetoconductance (MC) peak at *B=*0 T (refs 24, 25). In small, fully phase-coherent devices like ours, however, WAL is eclipsed by conductance fluctuations originating from the random interference of electron waves^25^,^26^. Indeed, in Fig. 2a, which shows the MC as a function of *V*_*g*_ and *B*, an enhancement in conductance at *B=*0 T is only faintly visible. No special feature at *B=*0 T can be detected by looking at a single MC curve measured at a fixed value of *V*_*g*_ (see, for example, the top curve shown in Fig. 2b measured at *V*_*g*_=–25 V). The random conductance fluctuations, whose reproducibility is shown in Fig. 2c, can be suppressed through an ensemble averaging procedure in which MC traces measured at different *V*_*g*_ values are averaged^25^. The *V*_*g*_ spacing should be chosen to shift the Fermi level by Thouless energy of the system. It is expected that the root mean square amplitude of the fluctuations decreases as *N*^−1/2^ (*N* is number of uncorrelated MC traces used to calculate the average), eventually making the sharp conductance peak at *B=*0 T due to WAL visible, if the strength of SOI is sufficient. This is indeed what the experiments show (Fig. 2b,d,e).

**Figure 3:**
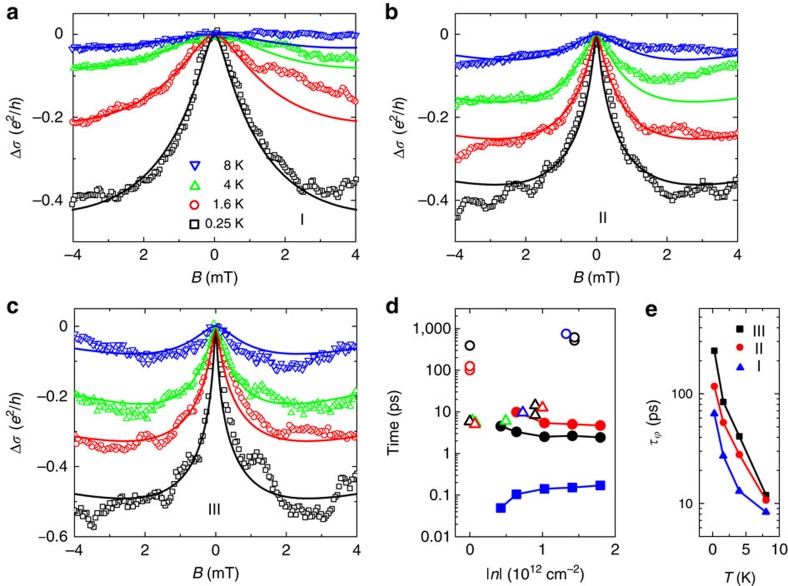
Low-temperature WAL in graphene on WS_2_. (**a**–**c**) Ensemble-averaged MC curves (symbols) obtained from measurements performed in different ranges of *V*_*g*_ (I, II and III, respectively), at several different temperatures below 8 K. The square MC Δ*σ*=*σ*(*B*≠0)–*σ*(*B*=0) clearly exhibits a peak at zero *B* in all *V*_*g*_ ranges, whose height decreases as temperature is increased from 250 mK to 8 K, the expected behaviour of WAL due to SOI. Solid lines show the best fits to equation (1) in the main text. (**d**) Carrier density dependence of the relevant characteristic times. The filled squares represent the elastic scattering time (*τ*) estimated from the conductivity of our device at zero *B*; the filled black (red) circles represent the spin relaxation time (*τ*_so_) extracted from the analysis of WAL (non-local spin-Hall effect). For comparison, open up-triangles represent the values of intervalley scattering time (*τ*_iv_) reported in the literature, and extracted from the analysis of weak-localization measured in device similar to ours on different substrates, such as SiO_2_ (black^27^ and red^29^), hBN (green^30^) and GaAs (blue^31^). Open circles represent *τ*_so_ obtained from spin-valve studies on pristine graphene on SiO_2_ (black^37^ and red^38^) and hBN (blue^40^). (**e**) Temperature dependence of the phase-coherence time (*τ*_*φ*_) of electrons in graphene-on-WS_2_ extracted from the analysis of WAL performed in this work, for different gate-voltage ranges. The data clearly exhibit an increase in *τ*_*φ*_ with lowering temperature.

We find that the WAL signal emerging from the ensemble average procedure is robust, and visible in the entire *V*_*g*_ range investigated. Its amplitude grows on lowering temperature *T* (Fig. 3a–c), and reaches ∼0.5 × *e*^2^*/h* at the largest negative *V*_*g*_ and *T*=250 mK (Fig. 3c), where *e* is electron charge and *h* is Planck's constant. The phenomenon is not observed in graphene on conventional substrates such as SiO_2_ (refs 27, 28, 29) (or hBN^30^ or GaAs^31^), where at sub-Kelvin temperatures only weak localization is measured. This remark is important because in graphene WAL can occur also in the absence of SOI, due only to the Dirac nature of its charge carriers^32^,^33^,^34^. The WAL originating from the Dirac nature of electrons, however, is unambiguously different from what we observe on WS_2_ substrates: it is seen only for *T*∼10 K or higher, and has small amplitude, because its observation requires the phase coherence time *τ*_*φ*_ to be shorter than the intervalley scattering time *τ*_iv_ (ref. 28). The observation of the low-temperature MC shown in Fig. 3, therefore, represents a direct, unambiguous demonstration of the presence of SOI in graphene on a WS_2_ substrate.

### Very short spin relaxation time in graphene on WS_2_

To analyse the MC data quantitatively, we use the theory of WAL in graphene that considers the effect of all possible symmetry-allowed SOI terms in graphene, and predicts the following dependence of the low-temperature MC^35^:


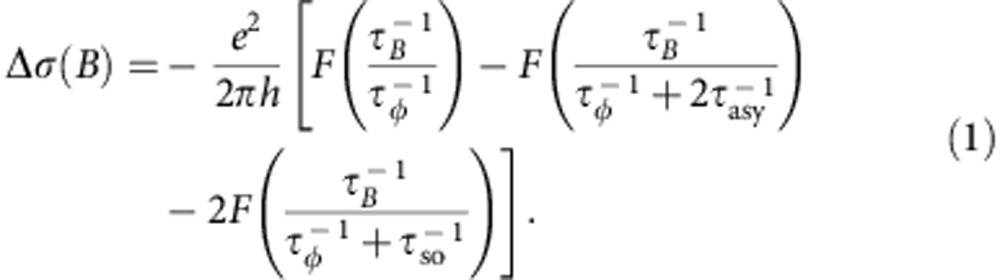


Here 
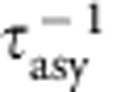
 is the rate of spin relaxation uniquely due to the SOI terms that break the *z*→*–z* symmetry (*z* being the direction normal to the graphene plane), 
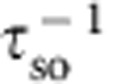
 is the total spin relaxation rate due to all SOI terms present, 
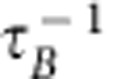
=4*DeB/ħ* (*D* is the carrier diffusion constant) and *F*(*x*)*=ln*(*x*)*+ψ*(1/2+1*/x*) with the digamma function *ψ*(*x*). In fitting the data, we constrain all characteristic times to be independent of temperature, except for *τ*_*φ*_, which increases on lowering *T*, as physically expected in the *T* range investigated. Equation (1) holds in the limit *τ*_*φ*_>>*τ*_iv_, which is the one physically relevant at low *T*, and reproduces the experimental results quantitatively (solid lines in Fig. 3a–c). The analysis allows us to obtain the relevant characteristic times *τ*_so_, *τ*_asy_ and *τ*_*φ*_, with a precision determined by the residual conductance fluctuations that are not perfectly removed by the ensemble averaging procedure. (These residual effects also cause *σ* to be non-perfectly symmetric on reversing *B*, because the conductivity is extracted from the conductance measured in a four-terminal configuration, which in fully phase coherent devices is in general not symmetric^25^.) We conservatively estimate the error on the characteristic times to be ∼50% in the worst case: although rather large as compared with what can be achieved in more established material systems, such an uncertainty is immaterial for all the considerations that will follow.

We find the spin-relaxation time to be *τ*_so_∼2.5–5 ps depending on the value of *V*_*g*_ (see the black filled circles in Fig. 3d). Comparable values (within experimental uncertainties) have been obtained on the same devices from the analysis of measurements of non-local resistance generated by spin-Hall and inverse spin-Hall effect^36^ (see red circles in Fig. 3d, Supplementary Fig. 3 and Supplementary Note 3 for details). The latter technique was used recently in refs 9, 10 to probe SOI in hydrogenated graphene and graphene on WS_2_ in devices analogous to ours (see the Supplementary Note 4 for a comparison). The value of *τ*_**asy_ is approximately three times larger than *τ*_so_, consistent with its physical meaning; the phase coherence time *τ*_*φ*_>>*τ*_so_, as it must be since a large WAL signal is observed (see Fig. 3e; *τ*_*φ*_ decreases on increasing *T*, as expected). This internal consistency of the hierarchy of characteristic times extracted from fitting the data with equation (1) supports the validity of our analysis. We conclude that in graphene at a WS_2_ interface *τ*_so_ is 100–1,000 times shorter than *τ*_so_ for pristine graphene on SiO_2_ (refs 37, 38) or hBN^39^,^40^ (shown with open circles in Fig. 3d), and that such a large difference in strength must be due to substantially stronger SOI. In contrast to what has been reported in recent studies of graphene on WS_2_ (see ref. 10 and Supplementary Note 4), the larger strength persists throughout the entire gate voltage range investigated.

Determining the precise nature of the WS_2_-induced SOI is not straightforward. A customary way to extract information is to identify the spin-relaxation mechanism by looking at how *τ*_so_ depends on *τ*, the transport scattering time. Finding that *τ*_so_ increases with increasing *τ* points to the so-called Elliot–Yafet relaxation mechanism (spin relaxation mediated by scattering at impurities)^41^,^42^, whereas if *τ*_so_ decreases with increasing *τ*, the Dyakonov–Perel mechanism (typical of systems with a strong band SOI) may be invoked^43^. In graphene on SiO_2_ or hBN substrates, previous work has shown that neither scenario convincingly accounts for the observations^8^, which has led to both phenomenological approaches to describe the experimental data^39^, and to the theoretical proposal of new spin-relaxation mechanisms specific to graphene^44^. For graphene on WS_2_, despite the much larger strength of SOI, the interpretation of spin relaxation within the canonical schemes poses similar problems: *τ*_so_ decreases slightly on increasing *τ* (see Fig. 3d), ruling out Elliot–Yafet as a dominant relaxation mechanism, but the dependence is much weaker than the one predicted by Dyakonov and Perel, *τ*_so_∝1*/τ*, so that the data are not satisfactorily described by this mechanism either. One interesting observation, however, can be made by comparing the spin-relaxation time *τ*_so_, with the intervalley scattering time *τ*_iv_ obtained from the analysis of weak localization in graphene on SiO_2_ (refs 27, 29), hBN^30^ and GaAs^31^. Literature values of *τ*_iv_ are shown with empty triangles in Fig. 3d: they are surprisingly narrowly distributed—they all fall within a factor of 2—if we consider that experiments have been performed by different groups and that different substrate materials are used. The values of *τ*_iv_ match well (again, within a factor of 2 or better) with *τ*_so_ obtained for graphene on WS_2_. This close correspondence between two *a priori* unrelated quantities is remarkable: it strongly suggests that the microscopic processes responsible for scattering between the two valleys are the same processes that cause spin flip in graphene on WS_2_.

### *Ab initio* calculations of interface-induced SOI

**Figure 4:**
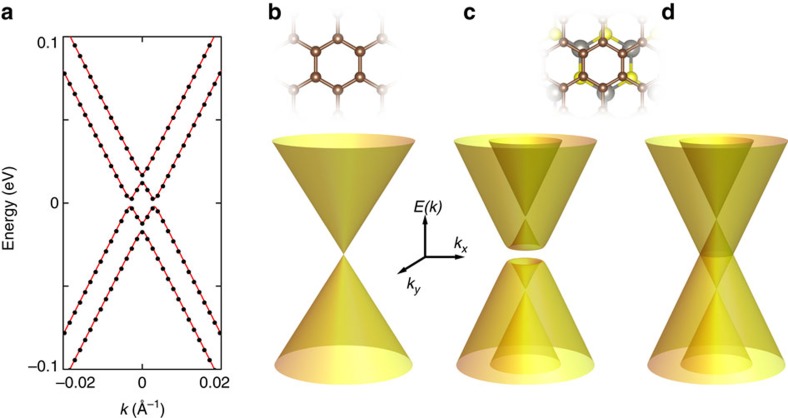
Low-energy band structure of graphene on WS_2_ near the K/K′ point. (**a**) Comparison of the result of the *ab initio* calculations with the continuum model Hamiltonian discussed in the main text. The black dots represent the low-energy dispersion relation for graphene on WS_2_ as obtained from our density functional theory calculations, which can be fit with excellent precision with the dispersion relation obtained from equation (2) (red lines). (**b**–**d**) Evolution of the low-energy dispersion relation of graphene as a function of SOI. (**b**) The usual Dirac cone for spin-degenerate charge carriers in isolated graphene close to the K/K′ point. (**c**,**d)** At an interface with a WS_2_ substrate, the dispersion relation is modified by the effect of the induced SOI. *Ab initio* calculations show that the low-energy Hamiltonian in equation (2) accurately describes the modifications to the band structure of graphene. Two SOI terms, with coupling constant *λ* and *λ*_*R*_, are induced by interfacial interactions. Our calculations indicate that *λ*∼5 meV and *λ*_*R*_∼1 meV. With these values the dispersion relation of electrons becomes the one shown in **c** that, at charge neutrality, corresponds to the band structure of an insulator (with non-trivial topological properties). The size of the gap is determined by the value of *λ*_*R*_ (as long as *λ*>>*λ*_*R*_). Since the gap is likely small in our devices as compared with electrostatic potential fluctuations, *λ*_*R*_ can be neglected in a first approximation, in which case the dispersion relation becomes the one shown in **d**.

To gain a better understanding of our experimental findings we have performed electronic structure calculations, to identify the dominant contributions to the WS_2_-induced SOI in graphene, and to verify that the large enhancement of the SOI strength we have discovered experimentally is indeed expected theoretically. Supercell electronic structure calculations were performed for a large number of crystal approximants to the incommensurate graphene-on-WS_2_ system. (See Supplementary Fig. 4 and Supplementary Note 5 for details.) The results demonstrate that hybridization between graphene and substrate orbitals adds SOI terms both to the graphene π-band Hamiltonian and to the π-band disorder Hamiltonian. For each approximant π-band states appear inside the WS_2_ gap *E*_*g*_ over a wavevector range of approximately *E*_*g*_*/ħv*_*F*_ surrounding both K and K′ Dirac points. Within this range π bands are accurately described by effective Hamiltonians of the form


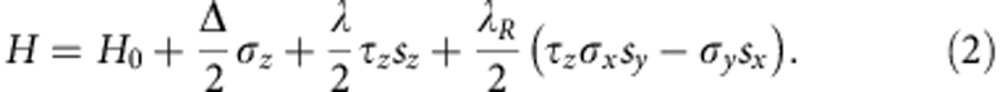


where **σ**=(σ_x_, σ_y_, σ_z_) is a Pauli matrix vector that acts on the sub lattice degree of freedom in graphene's Dirac continuum model Hamiltonian *H*_0_, ***s***=(s_x_, s_y_, s_z_) is a Pauli matrix vector that acts on spin and *τ*_*z*_=±1 for K and K′ valleys (the corresponding dispersion relations are illustrated in Fig. 4). All three substrate-induced interaction terms are time-reversal invariant and absent by inversion symmetry in isolated graphene sheets. They arise from hybridization between carbon *π* orbitals and strongly spin–orbit split tungsten *d* orbitals in both valence and conduction bands of WS_2_. Unlike the Hamiltonians that describe graphene on hBN^45^, *H* in equation (2) is translationally invariant. This is so because the lattice constant difference between graphene and WS_2_ is much larger than for the case of graphene and hBN. As a consequence, the moiré pattern period is short, and superlattice effects couple states near the K and K′ points to states far away in momentum and energy that are outside the range accessible to transport experiments and describable in terms of modified π bands.

In our calculations, the importance of a spatially random intervalley contribution to SOI in real structures, which are not commensurate, is inferred from the observation that the numerical values of the substrate interaction parameters (Δ, *λ* and *λ*_*R*_) depend on the supercell commensurability between graphene and WS_2_ triangular lattices (that is, the size of the approximant used in the calculation; see Supplementary Fig. 5). Physically, this dependence implies that spin-dependent terms that vary rapidly in space would be present even if each layer had a perfect, defect-free two-dimensional lattice. These terms can scatter graphene electrons between valleys via intermediate WS_2_ states. For this reason the continuum model of equation (2) should be supplemented by including random potential terms. (See the last paragraph of Supplementary Note 5 for a more complete explanation.) An estimate of the strength of these random potentials is given by the difference in the values of the parameters Δ, *λ* and *λ*_*R*_ obtained from calculations performed on approximants of different size (see Supplementary Fig. 5). This random spin-dependent substrate interaction provides a most plausible explanation for our finding that *τ*_so_ in graphene on WS_2_ is comparable to *τ*_iv_.

As a quantitative estimate of the parameters Δ, *λ* and *λ*_*R*_ in equation (2) we take the values obtained from the calculations performed on the largest supercells that we have considered. The 9:7 ratio between the WS_2_ and graphene lattice constants in these supercells is very nearly in perfect agreement with experiment. We find that the interaction parameters implied by this commensurability are independent of rigid relative translations between graphene and the substrate, providing further support for the translational invariance of equation (2). They are however sensitive to the separation between graphene and substrate layers, which has not yet been accurately determined experimentally, but should be near 3 Å like for graphene on hBN^46^. For such a separation our calculations give Δ≈0 meV, *λ*≈5 meV and *λ*_*R*_≈1 meV. The results illustrated in Supplementary Fig. 5 indicate that the value of *λ* is virtually the same for all approximants while larger relative fluctuations are found for Δ and *λ*_*R*_, implying that it is these terms that dominate the random spatial potential responsible for intervalley scattering. Even though at this stage it is difficult to establish the precise role of the two different SOI contributions (the modification of the bands and the random potentials) to the shortening of the spin-relaxation time extracted from the experiments, our results show that the two larger SOI parameters exceed the scale of the SOI in isolated graphene sheets by two to three orders of magnitude^3^,^4^,^5^, and that sizable spin-dependent intervalley scattering is present. We therefore can conclude that the results of our calculations are consistent with the experimental observations, pointing unambiguously to a strong enhancement of SOI due to interfacial interactions in graphene on WS_2_.

## Discussion

The general form of the Hamiltonian in equation (2), which yields a gap at charge neutrality because the Rashba term couples conduction and valence band states (compare Fig. 4c,d), deserves comment. It can be shown (see Supplementary Note 6) that when the Fermi energy lies in this gap and when *λ* is larger than Δ, the system becomes a topological insulator with gap larger by one to two orders of magnitude than that based on the intrinsic SOI in graphene originally proposed by Kane and Mele^2^. The result of our calculations for the largest supercell that we have considered (yielding Δ≈0 meV, *λ*≈5 meV and *λ*_*R*_≈1 meV) should be therefore be viewed as a prediction that graphene on WS_2_ is a topological insulator. This is an exciting conclusion, because a SOI strength of a few meV brings the possibility to realize a topologically insulating state in graphene closer to what can be realistically achieved experimentally. Care should be taken, however, because the precise values of the parameters Δ, *λ* and *λ*_*R*_ may depend on the details of the calculations scheme employed. In particular, even if in our calculations the SOI strength was always found to have a few meV scale irrespective of the approximations made, it is difficult to conclude that Δ>*λ* in all cases (see Supplementary Note 7, where we discuss calculations in which we let the distance between graphene and WS_2_ relax). At this stage it seems that a reliable determination of the parameters in equation (2) with sufficient precision to conclusively determine whether graphene on WS_2_ is a topological insulator or not will require more experiments. Specifically, this may be possible through a careful investigation of the Shubnikov–de Haas oscillations in very-high-mobility devices, capable of detecting a splitting in their frequency and of investigating its precise gate voltage dependence.

The increase in spin–obit interaction strength induced in graphene by proximity with WS_2_ that we observe for all investigated values of gate voltage—and hence position of the Fermi energy—is a large effect, close to two orders of magnitude. It directly shows the relevance of interfacial interactions. Demonstrating the ability to combine the control given by these interactions with the high electronic quality inferred from the experiments is a key result: although clearly more optimization is needed, finding that a drastic increase in SOI strength can be achieved without compromising the electronic quality of graphene offers the possibility to improve the system quality even further. To this end, strategies already exist that largely benefit from the expertise developed for graphene and hBN, such as encapsulating graphene between two WS_2_ crystals^16^, using different device assembly techniques to avoid contact between graphene and unwanted materials during fabrication^47^, and optimizing the device-cleaning procedures^48^. As indicated by both our experimental results and their theoretical modelling, these developments open a possible route to access experimentally topological states^49^ in graphene, and will play an essential role in improving our understanding of the spin dynamics in this system.

## Methods

### Device fabrication

Thin WS_2_ flakes were exfoliated onto a highly doped silicon substrate acting as a gate covered by a SiO_2_, from high-quality single crystals of WS_2_ grown by chemical vapour transport method. These exfoliated WS_2_ flakes were annealed at 200 degrees for 3 h in an inert atmosphere, after which a specific WS_2_ flake with atomically flat and clean surface was identified through imaging with an atomic force microscope. Transfer of graphene onto the selected WS_2_ flake was achieved by means of (by now) common techniques^15^. Similarly to the case of other artificial stacks of atomically thin crystals, bubbles were found to form after transfer of graphene (visible as black points in Fig. 1a)^30^. To minimize the effect of these bubbles on the transport experiments, graphene was etched into a Hall-bar geometry, after electrodes consisting of a Ti/Au bilayer (10/70 nm) were defined by means of electron-beam lithography, electron-beam evaporation and lift-off. No post-annealing steps or other cleaning processes to further improve the device quality were performed on the devices discussed here. All transport measurements were performed in a He^3^ Heliox system with a base temperature of 250 mK. We investigated in full detail two monolayer devices showing identical results. The data presented in the main text are taken from one of the devices, which has a width of *W*=2.5 μm, and three pairs of Hall probes (the longest distance between different pairs of Hall probes in this device is 5.5 μm, see Supplementary Fig. 3b). The thickness of WS_2_ is about 26 nm. Data from the second monolayer device, virtually identical to the first one, are shown in the Supplementary Note 1.

### *Ab initio* calculations

Fully relativistic density functional theory calculations were performed using the Vienna ab-initio Simulation Package with projector-augmented wave pseudopotentials under the generalized gradient approximation^50^,^51^. The graphene lattice constant was always kept at 2.46 Å. Ionic relaxations were performed for WS_2_ in 1 × 1 unit cells with lattice constants fixed to three different rational multiples (4/3, 5/4 and 9/7) of that of graphene. Supercells with different moiré periodicities were then constructed by repeating these unit cells correspondingly and by aligning the lattice vectors of WS_2_ with that of graphene without further ionic relaxation. The separation between graphene and WS_2_ was fixed to a number of different values ranging from 2.3 to 3.3 Å for each supercell. A Monkhorst-Pack *k*-point mesh^52^ of 6 × 6 × 1 was used for the 4 × 4 and 5 × 5 supercells (in terms of graphene lattice constant), and that of 3 × 3 × 1 was used for the 9 × 9 supercell. The plane wave energy cutoff was set to 400 eV in all calculations.

## Additional information

**How to cite this article:** Wang, Z. *et al*. Strong interface-induced spin-orbit interaction in graphene on WS_2_. *Nat. Commun.* 6:8339 doi: 10.1038/ncomms9339 (2015).

## Supplementary Material

Supplementary Information Supplementary Figures 1-5, Supplementary Notes 1-7 and Supplementary References
