# Supplementary material for: Strong interface-induced spin–orbit interaction in graphene on WS2
Source: Nat Commun. 2015 Sep 22;6:8339. doi: 10.1038/ncomms9339 (PMC4595714; doi:10.1038/ncomms9339)
Supplement: Supplementary Information — Supplementary Figures 1-5, Supplementary Notes 1-7 and Supplementary References [file ncomms9339-s1.pdf]

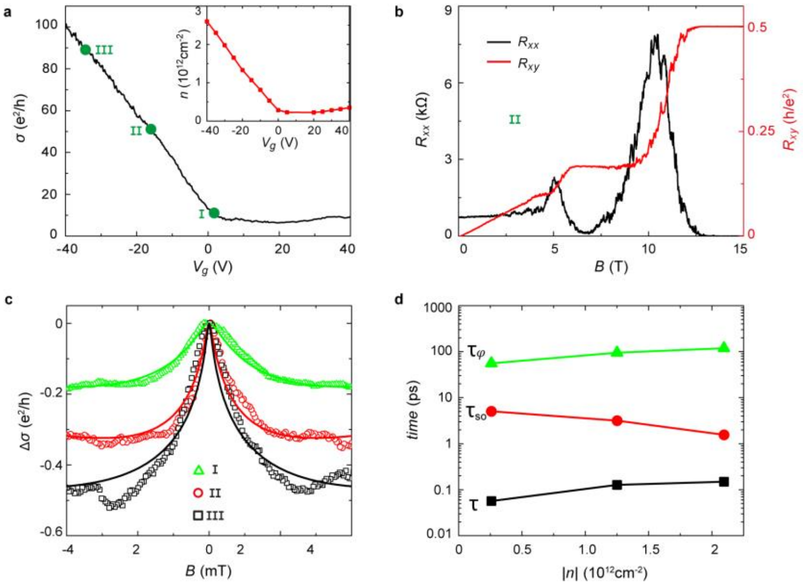

Supplementary Figure 1| Basic device characterization and observation of weak anti-localization in device 2 at 250 mK. (**a-b**) The basic characterization of device 2 shows a behavior virtually identical to that of the device discussed in the main text. The measured gate-voltage (V_g) dependence of the conductivity (σ) saturates at positive V_g (**a**; the inset further shows the concomitant saturation of n), and clear half-integer quantum-Hall effect is found for negative V_g (see panel **b**). (**c**) Ensemble-averaged magnetoconductance in the three different gate-voltage ranges (I: 5 ~ 0 V, II: -15 ~ -20 V, and III: -30 ~ -35 V) indicated in panel **a**. The sharp conductance peak at zero B –the characteristic feature of weak anti-localization– is clearly visible in all cases. (**d**) Density dependence of the estimated phase-coherence time (τ_{ϕ}; green up-triangles), spin-relaxation time (τ_{so}; red circles), and momentum relaxation time (τ; black squares).

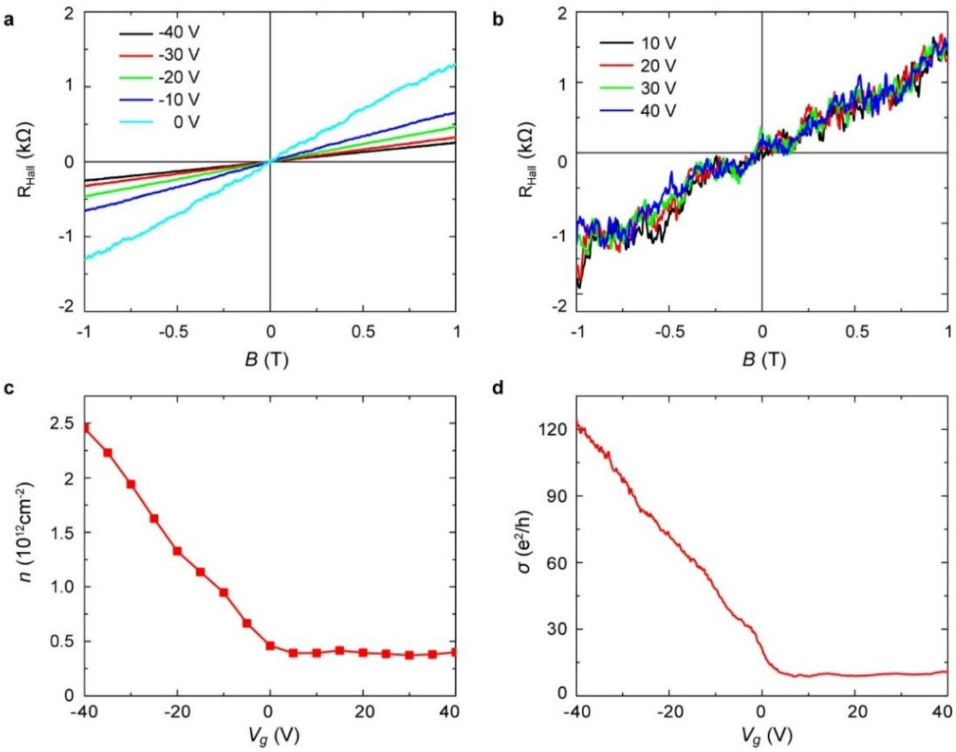

Supplementary Figure 2| Classical Hall effect and charge density as a function of back-gate voltage. (**a-b**) Hall resistance (R_{Hall}) as a function of magnetic field (B) measured at different values of gate-voltage, below and above 0 V. The slope, which measures the charge density accumulated in graphene, remains unchanged when the device is positively gate-biased. (**c**) Gate-voltage (V_g) dependence of the Hall density (n) extracted from the measurement of the Hall resistance. (**d**) Conductivity as a function of V_g, exhibiting a V_g dependence paralleling that of n shown in panel **c**.

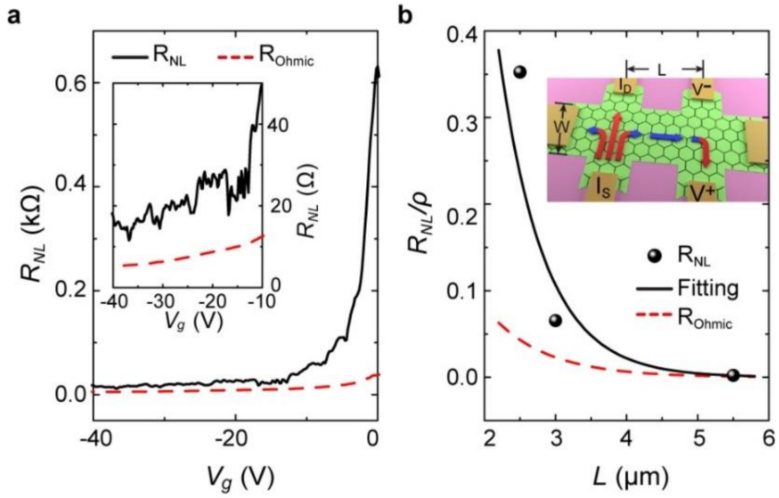

Supplementary Figure 3 | Non-local resistance measurement at 1.6 K. (**a**) V_g-dependence of the non-local resistance R_{NL} (black line) compared to the contribution from Ohmic transport (red broken line), $R_{ohm} = \rho e^{-\pi L/W}$ (with $L/W = 1.2$ in the present case). R_{NL} is larger than R_{ohm} in the entire V_g range explored. (**b**) At a fixed V_g = -27 V, the measured R_{NL} (black dots) exhibits a very rapid decay upon increasing L, consistent with an exponential behavior (quantitatively not consistent with the behavior expected for the Ohmic signal, represented by the red dashed line). As illustrated in the inset, $R_{NL} = V_{NL}/I_c$ is obtained by injecting a charge current I_c (red arrow) from source to drain contacts (denoted by I_S and I_D, respectively), and measuring the non-local voltage $V_{NL} = V^+ - V^-$ between two probes (denoted correspondingly) separated from the injecting contacts by a distance L.

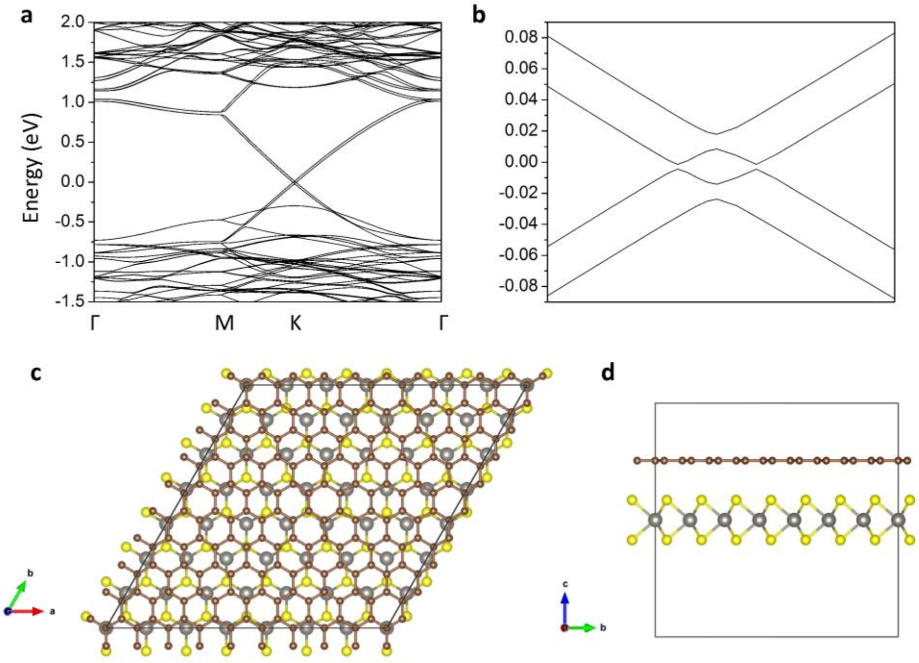

Supplementary Figure 4 | Band structure of graphene/WS_2 for short period commensurate moiré supercell. (**a**) band structure of a supercell in which 4×4-repeated monolayer WS_2 is lattice matched to 5×5-repeated graphene. In this calculation the separation between the layers was fixed at 2.45 Å. Calculations using a bilayer WS_2 substrate which preserves inversion symmetry have also been performed with qualitatively similar band structures for the graphene states inside the WS_2 band gap. The momentum labels refer to high-symmetry points in the supercell Brillouin-zone. (**b**) zoom view of the low-energy graphene-like bands inside the WS_2 band gap. (**c**) top and (**d**) side views of the moiré supercell for the 9:7 lattice constant ratio case.

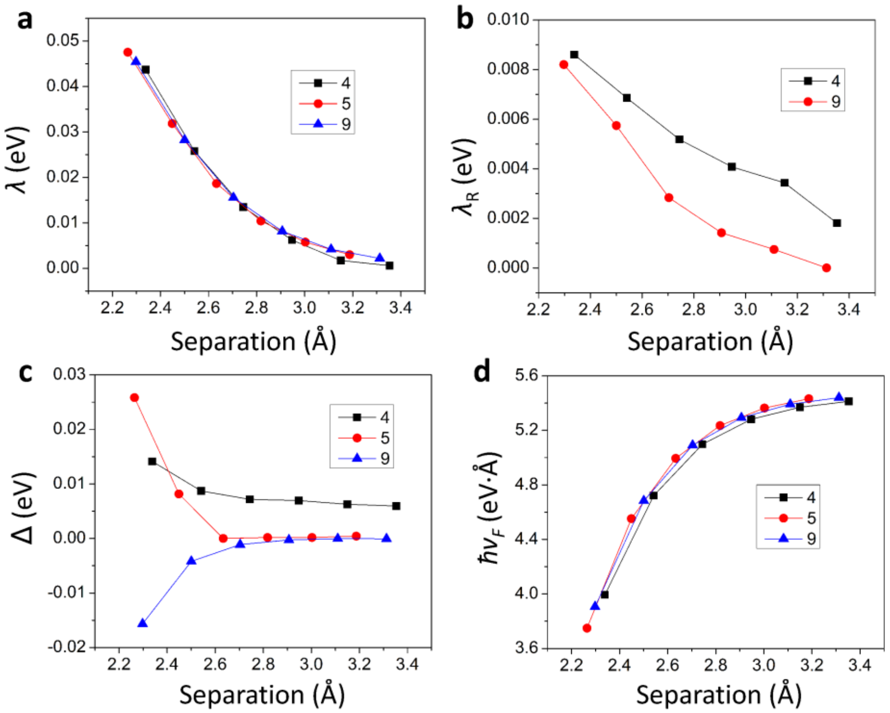

Supplementary Figure 5 | Coupling constants in the low-energy effective model of graphene on WS_2 obtained from DFT. (**a-d**) plot the dependence of λ, λ_R, Δ, and $\hbar v_F$ on the separation between graphene and WS_2 for three different commensurate moiré supercells. In ***b*** the case of the 5×5 supercell is not plotted since the splitting due to λ_R is too small in this case to enable a reliable estimate. Unlike λ and $\hbar v_F$, whose values collapse on the same curves for different supercell sizes, those of λ_R and Δ are apparently different for different supercells. The values of these terms are evidently sensitive to small changes in the average local coordination between graphene and WS_2. Because the lattice constant ratio in the 9:7 commensurate supercell calculations is very close to the experimental ratio, the parameters inferred from these calculations should provide a good representation of the experimental situation.

Supplementary Note 1: Weak anti-localization effect measured in different devices.

As discussed in detail in the main text, the observation of weak anti-localization (WAL) at low temperature unambiguously demonstrates the presence of strong SOI in graphene-on-WS_2. To illustrate the reproducibility of our observations, here we show data from a different device measured at the lowest temperature of our cryostat (T = 250 mK), which are virtually identical to those obtained from the device discussed in the main text. Supplementary Figure 1a shows that the conductivity varies linearly with V_g only for negative gate voltages, and the inset illustrates the V_g-dependence of n. The quantum Hall effect data in Supplementary Figure 1b clearly confirms that holes in the device behave as Dirac fermions[1]. Since the device dimension is similar to that of the device discussed in the main text, we reveal WAL by ensemble averaging the measured magnetoconductance around three different values of gate voltage; the results of the averaging are shown in Supplementary Figure 1c. It is apparent that a sharp conductance peak at zero magnetic field appears in all gate-voltage ranges explored, with an amplitude as large as ~$0.5e^2/h$ at the largest negative gate-voltages. The data are well fit by Eq. (1) of the main text[2] (see the continuous lines in Supplementary Figure 1c); the uncertainty in the fitting parameters originates from the effect of the residual random conductance fluctuations remaining after the averaging process. The characteristic times extracted from the fitting are shown in Supplementary Figure 1d, and exhibit values and trends virtually identical to those obtained from the device discussed in the main text.

Supplementary Note 2: Saturation of charge density in graphene-on-WS_2 at positive V_g occurring concomitantly with the conductivity saturation.

In the main text, we show that the conductivity (σ) of graphene-on-WS_2 devices saturates for V_g > ~8 V, because upon changing the gate voltage in this range, charges are accumulated at the WS_2/SiO_2 interface (where the carrier mobility is low) and not in graphene. Here, we confirm this conclusion by measuring the Hall effect in different gate-voltages ranges, to show that the Hall density (n) remains constant in the interval of V_g where σ saturates. Supplementary Figure 1a-b show the Hall resistance (R_{Hall}) as a function of magnetic field (B), measured for values of V_g below and above 0 V, respectively. It is apparent that the slope of this curve, which measures the charge density (n) accumulated in graphene, changes upon varying V_g below 0 V, and remains unchanged for positive V_g. A comparison between Supplementary Figure 1c and 1d, in which the carrier density and the conductivity are shown as a function of V_g between -40 V and 40 V, demonstrates that the saturation of n parallels the saturation of σ, as expected.

Supplementary Note 3: Non-local resistance as a signature of SOI.

We note that demonstrating the presence of strong SOI by measuring WAL effect at low temperatures, an entirely established method, has not been achieved previously in graphene. Related earlier work, including a recent one (further discussed in the section Supplementary Note 4) also focusing on graphene-on-WS_2 [3], has relied on another transport phenomenon, namely the measurement of non-local resistance (R_{NL}) generated through the combination of spin-Hall and inverse spin-Hall effect (often referred to as the non-local spin-Hall effect)[4]. This method works when the contribution to the non-local resistance due to this phenomenon is larger than the Ohmic contribution (which can be estimated to be $R_{ohm} = \rho e^{-\pi L/W}$, ρ is the resistivity and L/W is the device aspect ratio). The non-local signal due to the spin-Hall and inverse spin Hall effect decays exponentially away from the contacts used to inject current on a characteristic scale determined by the spin-relaxation length $\lambda_{so} = (D\tau_{so})^{1/2}$. Although our devices are not intentionally designed to optimally perform non-local

measurements, the multi-terminal Hall bar geometry nevertheless allows us to probe non-local signals. The results of these non-local measurements for $V_g < 0$ V (i.e., away from the V_g-region where the conductivity of graphene saturates) are shown in Supplementary Figure 3. It is apparent that R_{NL} in this regime is larger than R_{Ohm} (by approximately a factor of 2 to 3, depending on V_g, Supplementary Figure 3a) and that it decays very rapidly with increasing L, the distance between the contacts used to inject current and those used to detect voltage (Supplementary Figure 3b). The data are compatible with an exponential decay (black line in Supplementary Figure 3b) and using the formula $R_{NL} = \gamma^2 \rho W/2\lambda_{so} e^{-L/\lambda so}$ for the expected behavior of the non-local signal due to the spin-Hall effect (γ is the spin-Hall coefficient), we estimate values of τ_{so} (see the filled red dots in Fig. 3d of the main text) that are very close to those inferred from the analysis of WAL (the factor 2 difference is certainly compatible with the errors associated to the non-ideal device geometry for the analysis of non-local effect and with the precision of the WAL analysis; as mentioned in the main text, such level of uncertainty in τ_{so} does not affect the conclusions of our study).

Supplementary Note 4: Discussions of a recent study of SOI in graphene-on-WS_2.

In previous work by A. Avsar *et al.*[3] the role of spin-orbit interaction in graphene/WS_2 was studied by performing non-local transport measurements. (See Supplementary Note 3.) Although the present study and the earlier work agree in concluding that SOI is induced in graphene by the WS_2 substrate, there exist several important differences. In Supplementary Ref. 3, it is claimed that charge scattering by sulfur vacancies in the WS_2 substrate is the origin of strong SOI, not interfacial interactions with the substrate. *ab initio* calculations, used to support its claim, were also reported, showing no signature of SOI on the band structure of graphene-on-WS_2. The following comments are intended to shed light on the origin of these differences.

1. In Supplementary Ref. 3, experimental evidence for an enhanced SOI is found only for positive V_g, in the range where the measured conductance saturates, *i.e.* in the gate voltage range over which the transport properties of graphene cannot be tuned by the back gate. A. Avsar *et al.* also stated that there is no signature of WAL for negative V_g (see Supplementary Figure 10 in Supplementary Ref. 3 and the discussion related to it), a finding that was argued to support the consistency of their results. However, as clearly shown in our study, WAL in graphene-on-WS_2 devices is present throughout the investigated range for which the Fermi level is in the valence band (negative V_g). Our results also show that revealing the WAL signal requires a careful analysis. In particular, it is necessary to first eliminate the effect of random conductance fluctuations. In addition, in a high-mobility device (mobility of a few tens of thousands $cm^2V^{-1}S^{-1}$), WAL manifests itself in a signal visible only at very small magnetic field, of order a few milli-Tesla. Neither ensemble averaging nor measurements focusing on an appropriately small magnetic field range were shown in Supplementary Ref. 3.

2. In Supplementary Ref. 3, the conductance saturation observed for positive gate voltage is attributed to the presence of S vacancies in WS_2 close to graphene. Specifically, upon increasing the carrier density, the Fermi energy in graphene was claimed to align to defect states originating from the Sulfur vacancies. SOI was then claimed to result from interaction of carriers in graphene with vacancies in WS_2. We note that, because of

simple electrostatics, when charges can be accumulated in WS_2, they are accumulated at the interface closer to the gate, since this is the configuration of minimum electrostatic energy. Considering the accumulation of charges in WS_2 at the interface with graphene, therefore, does not seem a consistent interpretation and, consequently, an appropriate description of the origin of the induced SOI.

3. There also exist differences in the theoretical calculations. Even though the study of Supplementary Ref. 3 and our work use the same methods to calculate the band structure of graphene-on-WS_2, the results are notably different. In Supplementary Ref. 3, the calculation showed no signature of SOI in the band structure while in our study, SOI is clearly visible. As illustrated by Supplementary Figure 4, the absence of apparent SOI effects in Supplementary Ref. 3 might be due to the layer separations at which those calculations were performed.

Supplementary Note 5: Electronic Structure of Graphene on WS_2.

The role of spin-orbit coupling in the electronic structure of graphene on WS_2 can be addressed theoretically using the tools of electronic structure theory. This approach is complicated by the lattice constant mismatch between the two materials, and by the fact that the separation between layers may not be reliably predicted on the basis of purely theoretical considerations. To make progress we have performed electronic structure calculations (details are given in the Methods section) for super cells with three different ratios between the lattice constants of WS_2 and graphene, and for a wide range of layer separations. We find that in all cases a set of states whose wavefunctions that are strongly peaked in the graphene layer appear inside the WS_2 gap. The electronic structure of these states is always well described by a Hamiltonian in which the Dirac continuum model of isolated graphene is supplemented by three momentum and position independent substrate interaction terms,

$$H = H_0 + \Delta/2\sigma_z + \lambda/2\,\tau_z s_z + \lambda_R/2(\tau_z\sigma_x s_y - \sigma_y s_x) \quad (1)$$

Here H_0 is the spin-independent Dirac Hamiltonian of isolated graphene and $\boldsymbol{\sigma}$, τ_z, and $\boldsymbol{s}$ act respectively on its sublattice, valley, and spin degrees of freedom. The first two terms give rise to spin and valley dependent gapped massive Dirac bands which overlap in energy when $\lambda > \Delta$. The third term leads to avoided crossings at finite momentum when bands with different values of $\tau_z s_z$ overlap. Our *ab initio* numerical calculations showed no evidence of moiré periodicity effects like those that produce strong anomalies in electronic properties at four carriers per moiré period in the case of graphene on hexagonal Boron nitride with small relative orientation angles. A typical superlattice band structure is illustrated in Supplementary Figure 4. WS_2 and graphene share triangular Bravais lattices but WS_2 has a larger lattice constant (~3.16 Å) than that of graphene (2.46 Å). Supercells were constructed by choosing three different rational approximants to the lattice constant ratio, 4:3, 5:4, and 9:7. The 9:7 case (Supplementary Figure 4c and 4d) requires that calculations be performed for the largest supercells, but closely approximates the experimental lattice constant ratio. A previous study[3] reported on band calculations for the 4:3 lattice constant ratio.

Since τ_z $\boldsymbol{s}$ and σ_y are odd under time reversal, all three substrate interaction terms are time reversal invariant. Similarly all three terms are absent in an isolated graphene sheet because of inversion symmetry, which maps τ_z to $-\tau_z$ and

transforms the $\boldsymbol{\sigma}$ matrices like σ_x. Although we expect a term in the low energy model Hamiltonian that is proportional to $\tau_z\sigma_z s_z$, which *is* symmetry allowed even in the isolated graphene case[5], this term is evidently too weak for it to be manifested in the microscopic supercell calculations.

In Supplementary Figure 5 we plot coupling constants, obtained by fitting Supplementary Eq. 1 to *ab initio* supercell bands, as a function of the separation between graphene and WS_2 for 4:3, 5:4, and 9:7 commensurability. In each case the microscopic bands are in principle dependent on the position of the origin of the graphene Bravais lattice relative to the origin of the substrate Bravais lattice. We find however that these rigid translations have negligible effect on the graphene bands in practice. This finding supports the view that the spatial variation of the local coordination between the graphene sheet and the substrate does not lead to an important spatial variation in the effective Hamiltonian. Instead the orbitals of interest are influenced only by a spatially averaged effect of the substrate. When $\Delta \neq 0$ this averaging evidently still allows for a distinction between the graphene sheet sublattices but, as illustrated in Supplementary Figure 5 this effect is smaller for the experimentally relevant 9:7 lattice constant ratio and also smaller at the physically relevant layer separation. Experimentally the graphene/WS_2 separation has not yet been accurately determined, making it difficult to infer realistic values of the coupling parameters from the DFT calculations. Theoretically the weak van der Waals coupling between graphene and WS_2 cannot be correctly accounted for by conventional density-functional-theory approximations like LDA or GGA. Several semi-empirical schemes for including dispersion forces in DFT have been proposed in the past decades, but still have limited predictive power. As an educated guess, we expect the graphene/WS_2 separation to be around 3Å based on measurements performed for graphene on h-BN substrates[6]. For this separation one can estimate from Supplementary Figure 5, using data from the largest supercell calculations, that $\Delta \approx 0$ meV, $\lambda \approx 5$ meV, and $\lambda_R \approx 1$ meV.

The low-energy effective Hamiltonian for a graphene on WS_2 or on any other insulating or semiconducting substrate can always be separated into band Hamiltonian and disorder contributions. The latter terms account for broken translational symmetry. Our effective Hamiltonian parameters are determined by fitting the dressed π-bands over the fraction (~ 1/4) of the supercell Brillouin zone (for the 5/4 supercell) where they reside inside the WS_2 gap, and therefore represent averages over areas containing about 16 supercells or around 400 graphene unit cells. Because they are insensitive for a given commensurate structure to rigid displacements of the substrate relative to the graphene sheet, we can conclude that they reflect the band Hamiltonian. Our calculations demonstrate however that these band parameters, including those for the spin-dependent terms, are dependent on the commensurate structure examined. Since the actual structure of graphene on WS_2 is likely incommensurate, this sensitivity will be manifested as a disorder contributions to the low-energy effective Hamiltonian.

Supplementary Note 6: Graphene-on-WS_2 as a possible topological insulator

Within a single valley, Hamiltonian Supplementary Eq. 1 has the same form as the low-energy Hamiltonian in Supplementary Ref. 7 that describes the quantum anomalous Hall (QAH) insulator phase of graphene under both a Zeeman field and Rashba spin-orbit coupling. The difference between Supplementary Eq. 1 and the Hamiltonian in Supplementary Ref. 7 is that the Zeeman-like field in the present case, which is supplied by the spin-orbit coupling term

proportional to λ, has opposite signs in the two valleys as required by time-reversal symmetry. Extrapolating from the ideas of Kane and Mele[5], one expects to obtain a topological insulator by combining two mutually time-reversed copies of a QAH insulator. To verify that Supplementary Eq. 1 indeed describes a topological insulator, we follow Ref. 8 by calculating the Z_2 topological invariant using

$$Z_2 = \frac{1}{2\pi}\left[\oint_{\partial B+} d\boldsymbol{k}\cdot\boldsymbol{A}(\boldsymbol{k}) - \int_{B+} d^2k\,\Omega_z(\boldsymbol{k})\right] \bmod 2 \tag{2}$$

where B^+ and ∂B^+ are respectively a half Brillouin zone and its boundary, and $\boldsymbol{A}(\boldsymbol{k})$ and $\Omega_z(\boldsymbol{k})$ are respectively the Berry connection and the Berry curvature summed over filled bands. Z_2=1 for a topological insulator and Z_2=0 for a trivial insulator. In the present case the half Brillouin zone can be identified with an area around a single valley and a boundary that is sufficiently far from the centre of the valley. We calculated the Z_2 number for λ much larger than λ_R and Δ and found that Z_2 is always 1. Therefore in the ideal situation in which the low energy physics of the graphene/WS_2 system is completely determined by Supplementary Eq. 1 the system should be a topological insulator. It is worth noting is that in the original Kane-Mele model the topologically nontrivial gap is opened by the intrinsic spin-orbit coupling of graphene which preserves inversion symmetry, whereas in the present model the gap is opened by two spin-orbit coupling terms, both of which break inversion symmetry and are absent in freestanding graphene.

Supplementary Note 7: Distance between graphene and WS_2 calculated with vdW functional

As the distance between graphene and WS_2 substrate is not experimentally determined yet, in this article we take it to be around 3Å (distance measured between graphene and h-BN[6]) and conclude strong SOI in the system. To avoid serious bias on it, we also relax the graphene-WS_2 distance with van der Waals functionals though this method has limited predictive power. We performed relaxation of the 5:4 graphene/WS_2 supercell using the optB86b-vdW functional[9] implemented in VASP[9-11], with a k-point mesh of 7×7×1 and the stopping criterion being the force on each atom smaller than 10^{-3} eV/Å. We found the graphene-WS_2 separation increases to 3.36 Å, with a slight corrugation of the graphene sheet of 0.05 Å at its maximum. We have also tried other van der Waals functionals and found that they give graphene-WS_2 separations different from one another with a variation of 0.3 Å. Despite the larger graphene-WS_2 separation and the graphene corrugation, we found the DFT band structure for the graphene can still be well fitted by the effective Hamiltonian Supplementary Eq. 1. The spin orbit coupling parameter λ is 1.7 meV, still two orders of magnitude larger than that in freestanding graphene. However, the sublattice splitting Δ also increases to be comparable with λ, mainly due to the slight corrugation of the graphene which enhances local matching between the graphene and the WS_2 lattices and hence the sublattice splitting. Since the effective Hamiltonian Supplementary Eq. 1 is a topological insulator only when $\Delta<\lambda$, the closeness between the fitted values of Δ and λ makes the topological character of the graphene more delicate. However, as these values are sensitively dependent on the graphene-WS_2 separation and the supercell periodicity, which cannot be reliably determined from first principles, whether the graphene/WS_2 system is a topological insulator or not should best be left for future experimental verification. Nevertheless we stress that the much larger spin-orbit splitting than that in freestanding graphene is still robust.

Supplementary References:

1 Castro Neto, A. H., Guinea, F., Peres, N. M. R., Novoselov, K. S. & Geim, A. K. The electronic properties of graphene. *Rev. Mod. Phys.* **81**, 109-162 (2009).

2 McCann, E. & Fal'ko, V. I. $z \rightarrow -z$ Symmetry of Spin-Orbit Coupling and Weak Localization in Graphene. *Phys. Rev. Lett.* **108**, 166606 (2012).

3 Avsar, A. *et al.* Spin-orbit proximity effect in graphene. *Nat. Commun.* **5**, 4875 (2014).

4 Abanin, D. A., Shytov, A. V., Levitov, L. S. & Halperin, B. I. Nonlocal charge transport mediated by spin diffusion in the spin Hall effect regime. *Phys. Rev. B* **79**, 035304 (2009).

5 Kane, C. L. & Mele, E. J. Quantum spin Hall effect in graphene. *Phys. Rev. Lett.* **95**, 226801 (2005).

6 Haigh, S. J. *et al.* Cross-sectional imaging of individual layers and buried interfaces of graphene-based heterostructures and superlattices. *Nat. Mater.* **11**, 764-767 (2012).

7 Tse, W.-K., Qiao, Z., Yao, Y., MacDonald, A. H. & Niu, Q. Quantum anomalous Hall effect in single-layer and bilayer graphene. *Phys. Rev. B* **83**, 155447 (2011).

8 Fu, L. & Kane, C. L. Time reversal polarization and a Z_2 adiabatic spin pump. *Phys. Rev. B* **74**, 195312 (2006).

9 Klimeš, J., Bowler, D. R. & Michaelides, A. Van der Waals density functionals applied to solids. *Phys. Rev. B* **83**, 195131 (2011).

10 Dion, M., Rydberg, H., Schröder, E., Langreth, D. C. & Lundqvist, B. I. Van der Waals Density Functional for General Geometries. *Phys. Rev. Lett.* **92**, 246401 (2004).

11 Román-Pérez, G. & Soler, J. M. Efficient Implementation of a van der Waals Density Functional: Application to Double-Wall Carbon Nanotubes. *Phys. Rev. Lett.* **103**, 096102 (2009).
